# Low bone mineral density due to secondary hyperparathyroidism in the *Gla^tm^Tg(CAG‐A4GALT)* mouse model of Fabry disease

**DOI:** 10.1096/fba.2019-00080

**Published:** 2020-06-10

**Authors:** Hiroki Maruyama, Atsumi Taguchi, Mariko Mikame, Hongmei Lu, Norihiro Tada, Muneaki Ishijima, Haruka Kaneko, Mariko Kawai, Sawako Goto, Akihiko Saito, Riuko Ohashi, Yuji Nishikawa, Satoshi Ishii

**Affiliations:** ^1^ Department of Clinical Nephroscience Niigata University Graduate School of Medical and Dental Sciences Niigata Niigata Japan; ^2^ Laboratory of Genome Research Research Institute for Diseases of Old Age Juntendo University Graduate School of Medicine Bunkyo‐ku Tokyo Japan; ^3^ Department of Medicine for Orthopaedics and Motor Organ Juntendo University Graduate School of Medicine Bunkyo‐ku Tokyo Japan; ^4^ Department of Pharmacology Osaka Dental University Hirakata Osaka Japan; ^5^ Department of Applied Molecular Medicine Niigata University Graduate School of Medical and Dental Sciences Niigata Niigata Japan; ^6^ Histopathology Core Facility Faculty of Medicine Niigata University Niigata Niigata Japan; ^7^ Division of Tumor Pathology Department of Pathology Asahikawa Medical University Asahikawa Hokkaido Japan; ^8^ Department of Matrix Medicine Faculty of Medicine Oita University Yufu Oita Japan; ^9^ Biochemical Laboratory GlycoPharma Corporation Oita Oita Japan

**Keywords:** 24‐hydroxylase, bone histomorphometry, osteomalacia, parathyroid hormone, renal phosphate wasting

## Abstract

Low bone mineral density (BMD)—diagnosed as osteoporosis or osteopenia—has been reported as a new characteristic feature of Fabry disease; however, the mechanism underlying the development of low BMD is unknown. We previously revealed that a mouse model of Fabry disease [*Gla^tm^Tg(CAG‐A4GALT)*] exhibits impaired functioning of medullary thick ascending limb (mTAL), leading to insufficient Ca^2+^ reabsorption and hypercalciuria. Here, we investigated bone metabolism in *Gla^tm^Tg(CAG‐A4GALT)* mice without marked glomerular or proximal tubular damage. Low BMD was detected by 20 weeks of age *via* micro‐X‐ray‐computed tomography. Bone histomorphometry revealed that low BMD results by accelerated bone resorption and osteomalacia. Plasma parathyroid hormone levels increased in response to low blood Ca^2+^—not plasma fibroblast growth factor 23 (FGF‐23) elevation—by 5 weeks of age and showed progressively increased phosphaturic action. Secondary hyperparathyroidism developed by 20 weeks of age and caused hyperphosphatemia, which increased plasma FGF‐23 levels with phosphaturic action. The expression of 1α‐hydroxylase [synthesis of 1α,25(OH)_2_D_3_] in the kidney did not decrease, but that of 24‐hydroxylase [degradation of 1α,25(OH)_2_D_3_] decreased. Vitamin D deficiency was ruled out as the cause of osteomalacia, as plasma 1α,25(OH)_2_D_3_ and 25(OH)D_3_ levels were maintained. Results demonstrate that secondary hyperparathyroidism due to mTAL impairment causes accelerated bone resorption and osteomalacia due to hyperphosphaturia and hypercalciuria, leading to low BMD in Fabry model mice.

**Abbreviations**
1,25(OH)_2_D_3_: 1α,25‐dihydroxyvitamin D_3_
25(OH)D_3_: 25‐hydroxyvitamin D_3_
Abs: antibodies
ALP: alkaline phosphatase
BMD: bone mineral density
BW: body weight
CD: collecting duct
CKD: chronic kidney disease
Cr: creatinine
CrCl: creatinine clearance
CTX‐I: C‐terminal telopeptide of type I collagen
CYP24A1: 24‐hydroxylase
CYP27B1: 1α‐hydroxylase
DCT: distal convoluted tubule
DEXA: dual‐energy X‐ray absorptiometry
FE: fractional excretion
FGF‐23: fibroblast growth factor 23
Gb3: globotriaosylceramide
mTAL: medullary thick ascending limb
P1NP: procollagen type I N‐terminal propeptide
PCT: proximal convoluted tubule
PST: proximal straight tubule
PT: proximal tubule
PTH: parathyroid hormone
PTH1R: parathyroid hormone 1 receptor
SLC34A1: solute carrier family 34 member 1
WT: wild‐type
μCT: micro‐X‐ray‐computed tomography

## 1 INTRODUCTION

Fabry disease is an X‐linked hereditary disease caused by mutations in *GLA*, which encodes the lysosomal enzyme α‐galactosidase A^1^; approximately 840 *GLA* mutations have been identified.^2^ Fabry disease targets multiple organs.^3^ The disease is characterized by systemic accumulation of glycosphingolipids, especially globotriaosylceramide (Gb3), in the lysosomes of various cell types.^4^ It can also be categorized into classic or late‐onset types based on the presence or absence of early classic manifestations—*ie*, acroparesthesia, clustered angiokeratoma, and cornea verticillata—and the type of *GLA* mutation.^3^ In 2005, low bone mineral density (BMD) was first reported as a previously unrecognized manifestation of Fabry disease in male patients with classic Fabry disease,^5^ and subsequent studies have highlighted the risk of low BMD in patients with classic Fabry disease.^6^, ^7^, ^8^ In these reports,^5^, ^6^, ^7^, ^8^ low BMD was diagnosed as osteoporosis and osteopenia based only on dual‐energy X‐ray absorptiometry (DEXA). However, the precise pathological diagnosis and the mechanisms underlying bone disease observed in patients with Fabry disease remain unknown.

Although BMD measurements are used in the diagnosis and management of osteoporosis, DEXA cannot distinguish between osteoporosis and osteomalacia.^9^ Osteoporosis and osteopenia are characterized by low bone mass.^10^ According to the World Health Organization, osteopenia is defined as having a BMD T‐score of between −1.0 and −2.5 (*ie*, 1.0–2.5 standard deviations below the mean), whereas osteoporosis has a T‐score below −2.5.^10^ Meanwhile, osteomalacia is defined as a mineralization defect caused by disorders that lead to decreased bone mineralization.^11^ Osteomalacia also results in low BMD^12^—due to decreased calcified bone volume—as well as increased osteoid volume and preserved total bone volume. By contrast, osteoporosis and osteopenia exhibit normal osteoid volume and decreased total bone volume.^11^, ^13^ Thus, the pathophysiology and treatment are completely different between these bone disorders. Osteomalacia is diagnosed histologically, and bone histomorphometry is essential for establishing a definitive diagnosis.^10^ A bone biopsy is therefore the only method capable of diagnosing osteomalacia.^13^ Osteomalacia is mainly caused by vitamin D deficiency and renal calcium (Ca) or phosphate (P) wasting, which is characterized by an increased filtered P load due to hyperphosphatemia (excess excretion of P) or decreased proximal tubular P reabsorption through solute carrier family 34 member 1 (SLC34A1) by the phosphaturic action of parathyroid hormone (PTH) and fibroblast growth factor 23 (FGF‐23).^12^

*Gla^tm^* mice do not develop Fabry disease due to a lower accumulation of Gb3 than that observed in humans.^14^ We had previously crossbred asymptomatic *Gla^tm^* mice^15^, ^16^ with transgenic mice expressing human Gb3 synthase (A4GALT) to generate the *Gla^tm^Tg(CAG‐A4GALT)* symptomatic Fabry mouse model that exhibits polyuria and renal dysfunction without any remarkable glomerular damage and provided the first clear evidence that Gb3 accumulation is the primary cause of Fabry disease.^17^ Recently, we employed the *Gla^tm^Tg(CAG‐A4GALT)* Fabry model mice with polyuria and demonstrated that the medullary thick ascending limbs (mTALs) were the primarily affected tubules and that TAL dysfunction—an inability to concentrate urine as a result of decreased expression of the core molecules Na^+^‐K^+^‐ATPase, uromodulin, and Na^+^‐K^+^‐2Cl^−^ cotransporter—induced water‐ and salt‐loss phenotypes.^18^ TALs play a critical role in divalent cation (Ca^2+^ and Mg^2+^) homeostasis by reabsorption *via* paracellular transport.^19^ Indeed, *Gla^tm^Tg(CAG‐A4GALT)* mice exhibit higher fractional excretion (FE) and daily urinary excretion of divalent cations compared with that in wild‐type (WT) mice by 20 weeks of age.^18^ Thus, the possibility that these findings may have an adverse effect on bone metabolism has been considered.

The TAL is also associated with the regulation of vitamin D endocrine system. 1α,25‐Dihydroxyvitamin D_3_ [1,25(OH)_2_D_3_] plays a vital role in Ca^2+^ metabolism and normal bone growth. 1α‐Hydroxylase (CYP27B1) and 24‐hydroxylase (CYP24A1) are enzymes belonging to the mitochondrial cytochrome P450 family that are responsible for the synthesis and degradation of 1,25(OH)_2_D_3_, respectively. CYP27B1^20^, ^21^ and CYP24A1^21^, ^22^ are expressed even in the distal tubules—the TAL and distal convoluted tubule (DCT)—the collecting duct (CD), and proximal tubule (PT). Therefore, the expression of these enzymes may be reduced in *Gla^tm^Tg(CAG‐A4GALT)* mice.

Although osteoporosis cannot be diagnosed as the cause of low BMD without bone histomorphometric analysis, a bone biopsy examination of low BMD in patients with Fabry disease has not yet been performed.^6^, ^7^, ^8^, ^9^ To determine the underlying cause of low BMD in Fabry disease, we herein investigated the bone status of mTAL‐impaired *Gla^tm^Tg(CAG‐A4GALT)* mice by measuring BMD and performing bone histomorphometric analysis, biochemical assessment of hormonal and other factors that regulate renal Ca and P handling, and by immunohistochemistry on kidney samples.

## 2 MATERIALS AND METHODS

### 2.1 Animals

The C57BL/6J‐*Gla^tm1kul^Tg(CAG‐A4GALT)* mouse line^17^ was generated by crossbreeding C57BL/6J‐*Tg(CAG‐A4GALT)* mice containing the *A4GALT* transgene at a single allele with homozygous *Gla* knockout C57BL/6J;129S4‐*Gla^tm1kul^* mice.^15^ Hereafter, we would use the following abbreviations: *Tg(CAG‐A4GALT)* for C57BL/6J‐*Tg(CAG‐A4GALT)*, *Gla^tm^* for C57BL/6J;129S4‐*Gla^tm1kul^*, and *Gla^tm^Tg(CAG‐A4GALT)* for C57BL/6J‐*Gla^tm1kul^Tg(CAG‐A4GALT)*. Both *Gla^tm^* and *Gla^tm^Tg(CAG‐A4GALT)* mice were backcrossed with the C57BL/6J strain mice more than six times. *Tg(CAG‐A4GALT)* mice were generated using the C57BL/6J strain mice. Thus, the genetic background of *Gla^tm^*, *Tg(CAG‐A4GALT)*, and *Gla^tm^Tg(CAG‐A4GALT)* mice is aligned to the C57BL/6J strain. WT mice (C57BL/6J) were purchased from Charles River Laboratories Japan (Yokohama, Japan). Mice were raised under standard laboratory conditions of 24 ± 2°C, and 50%–60% humidity under, and a 12/12‐hour light–dark cycle and were allowed ad libitum access to tap water and commercial standard rodent chow (CE‐2) containing 1.20% Ca, 1.08% P, and 240 IU/100 g vitamin D_3_ (Clea Japan, Tokyo, Japan). Mouse lines were genotyped by PCR amplification of *A4GALT,* as previously described.^17^ Female mice were excluded from the experiments as there is a possibility that the phenotype would get affected by pregnancy. All animal experiments were reviewed by the Institutional Animal Care and Use Committee and approved by the Presidents of Niigata University, Japan (permit numbers H21Niigata Univ. Res. 69, H25‐111 Niigata Univ. Res. 255‐1, H27‐182 Niigata Univ. Res. 323‐6, Japan), and Oita University, Japan (permit number P006002, Japan).

### 2.2 Body weight and tibia length measurement

Body weight (BW) was measured using a CS 200X balance (Ohaus, Pine Brook, NJ, USA), while tibia length was measured with an Absolute Super Caliper 500‐774 (Mitutoyo, Kawasaki, Japan).

### 2.3 Micro‐X‐ray‐computed tomography analysis

The BMD of the cortical bone, trabecular bone, and total bone were measured in an area 0.2–2 mm proximal to the growth plate of the distal femoral bone by micro‐X‐ray‐computed tomography (μCT) (Latheta LCT‐200; Hitachi Aloka Medical, Tokyo, Japan). The average value was determined based on twenty 48‐μm‐thick slices from a cross‐section of the femur. Image data were quantified using an automated image analysis system (Latheta software; Hitachi Aloka Medical).

### 2.4 Bone histomorphometry

To perform bone histomorphometry, 20‐week‐old male mice were double‐labeled with subcutaneous injections of calcein (20 mg/kg BW; Sigma‐Aldrich, St Louis, MO, USA) 5 and 2 days before euthanasia. The femurs of each mouse were removed and fixed with 70% ethanol. The femurs were then trimmed to remove the muscle, stained with Villanueva bone stain for 5 days, dehydrated in graded concentrations of ethanol, and embedded in methyl‐methacrylate (Wako Chemical, Kanagawa, Japan) without decalcification.^23^ Frontal plane sections (5‐μm‐thick) of the distal femur were cut using a microtome (RM2255; Leica Biosystems, Wetzlar, Germany). The cancellous bone in the secondary spongiosa was measured (located 250 μm from the epiphyseal growth plate and 250 μm from the endocortical surface). Histomorphometric parameters^24^ were measured at the Ito Bone Science Institute (Niigata, Japan) in a blinded fashion using a semiautomatic image analyzing system (System Supply, Ina, Japan) and a fluorescence microscope (BX‐51; Olympus, Tokyo, Japan). Villanueva Goldner staining was performed at the Kureha Special Laboratory Co., Ltd. (Iwaki, Japan). The samples were imaged using an All‐in‐One Fluorescence Microscope (BZ‐X700; Keyence, Osaka, Japan).

### 2.5 Blood analysis

Mice employed in the present study for the analysis of blood Ca^2+^, and plasma P and alkaline phosphatase (ALP) levels were the same as those used in a previous study.^18^ However, these data have been described for the first time in the present study. Blood Ca^2+^ was determined in whole blood using an i‐STAT analyzer (Abbott, Tokyo, Japan). Plasma was obtained by centrifuging whole blood at 500 × *g* for 10 minutes. Plasma P and ALP levels were measured by Oriental Yeast Co., Ltd. (Nagahama, Japan). Plasma procollagen type I N‐terminal propeptide (P1NP) levels were measured using the Mouse P1NP Enzyme‐Linked Immunosorbent Assay (ELISA) Kit (E‐EL‐M0233; Elabscience, Houston, TX, USA) with a detection threshold of 56.25 pg/ml. The levels of C‐terminal telopeptide of type I collagen (CTX‐I) were measured using the RatLaps™ (CTX‐I) EIA Kit (AC‐06F1; Immunodiagnostic Systems, Tyne and Wear, UK) with a detection threshold of 4.5 ng/ml. Intact PTH levels in the plasma were assayed using the Mouse PTH 1–84 ELISA Kit (Quidel, San Diego, CA, USA) with a detection threshold of 32 pg/ml. Plasma FGF‐23 levels were assayed using the FGF‐23 ELISA Kit (Kainos, Tokyo, Japan) with a detection threshold of 3 pg/ml. Plasma PTH and FGF‐23 measurements were performed using a SpectraMax Plus 384 Microplate Reader (Molecular Devices, Sunnyvale, CA, USA) at 450 nm and were quantified by SoftMax Pro Software 5.2 (Molecular Devices). Plasma 1,25(OH)_2_D_3_ and 25‐hydroxyvitamin D_3_ [25(OH)D_3_] levels were measured by liquid chromatography‐electrospray ionization‐tandem mass spectrometry by the Oriental Yeast Co., Ltd., with detection thresholds of 10 pg/ml for 1,25(OH)_2_D_3_ and 0.1 ng/ml for 25(OH)D_3_. Measurements were performed with an API‐5000 triplestage mass spectrometer (SCIEX, Framingham, MA, USA) equipped with an electrospray ionization ion source (SCIEX) and Agilent 1290 Infinity LC system (Agilent Technologies, Santa Clara, CA, USA).

### 2.6 Creatinine clearance

Plasma and urine creatinine (Cr) levels have been measured and described in our previous study.^18^ Using these data, we newly analyzed creatinine clearance (CrCl) in the present study. CrCl was calculated according to the formula: CrCl = ([Cr]_24‐hours urine_ × 24‐hours urine volume)/[Cr]_plasma_. Data were normalized to a BW of 25 g.^25^

### 2.7 Urine analysis

Urine samples used in the present study for urine P excretion analysis were the same as those analyzed in our previous study.^18^ However, we describe urine P excretion for the first time in the present study. Mice were kept in metabolic cages for 24‐hour urine collection. Urine P and Cr levels were analyzed by Oriental Yeast Co., Ltd. The FE of a solute X (FE_x_) was calculated according to the formula: (FE_x_) = ([X]_24‐hours urine_ × 24‐hours urine volume)/([X]_plasma_ × CrCl) × 100. Urine samples were collected from mice in metabolic cages for 24 hours and stored at −80°C until further use. Samples were centrifuged at 3500 × *g* at 4°C for 15 minutes, after which the supernatants were collected for ELISA.

### 2.8 Immunohistochemistry

Kidneys were transversely cut, fixed in 10% neutral buffered formalin, and embedded in paraffin. Deparaffinized sections (3 μm) were prepared by routine procedure. Immunohistochemical staining was performed after antigen retrieval. Anti‐SLC34A1 (NBP2‐13328; Novus Biologicals, Littleton, CO, USA), anti‐CYP27B1 (ABN182; EMD Millipore, Billerica, MA, USA), anti‐CYP24A1 (OABB02030; Aviva Systems Biology, San Diego, CA, USA), and anti‐parathyroid hormone 1 receptor (PTH1R) (HPA007978; Merck KGaA, Darmstadt, Germany) were combined with secondary Ab N‐Histofine simple stain mouse MAX‐PO (R) (414341; Nichirei, Tokyo, Japan). Anti‐megalin (provided by coauthor Dr Saito) was combined with secondary Ab biotinylated anti‐rabbit IgG (Vector Laboratories, Burlingame, CA, USA). Anti‐F4/80 (MCA497GA; AbD Serotec, Raleigh, NC, USA) was combined with secondary Ab N‐Histofine simple stain mouse MAX‐PO (rat) (414311; Nichirei). Counterstaining with hematoxylin was performed on all slides. Finally, samples were imaged using the BZ‐X700 microscope. Information on the Abs used in this study is provided in Table S1. For negative controls, immunohistochemistry was performed without primary Abs (Figure S1); no protein signals were detected, indicating that nonspecific binding of secondary Abs did not occur.

### 2.9 Western blot analysis

The DC Protein Assay (Bio‐Rad Laboratories, Hercules, CA, USA) was used to quantify protein concentrations, after which equal amounts of whole kidney homogenates were loaded onto the gel. Anti‐SLC34A1 (NBP2‐13328; Novus Biologicals), anti‐CYP27B1 (ABN182; EMD Millipore), anti‐CYP24A1 (OABB02030; Aviva Systems Biology), and anti‐glyceraldehyde‐3‐phosphate dehydrogenase (GAPDH) (G9545; Millipore Sigma, St. Louis, MO, USA) were combined with secondary Ab anti‐rabbit IgG (ab97051; Abcam, Cambridge, UK). Anti‐megalin (provided by coauthor Dr Saito) was combined with secondary Ab anti‐rabbit IgG (eBioscience, San Diego, CA, USA). Immunoblots were developed using the SuperSignal West Pico Chemiluminescent Substrate (Thermo Fisher Scientific, Waltham, MA, USA), scanned with an ImageQuant LAS 4000 mini (GE Healthcare Life Science, Piscataway, NJ, USA), and quantified using ImageQuant TL v.8.1 software (GE Healthcare Life Science). Information on regarding Abs used in this study is provided in Table S1.

### 2.10 Statistical analysis and graph preparation

At least 5 mice/group were used for all experiments, except for histological analysis; the group size was based on previous studies.^17^, ^18^ A power analysis was not performed to determine the sample size. We did not randomize samples or mice for experiments. For animal studies, no blinding was performed except in the case of bone histomorphometry. The Shapiro–Wilk test was used to test for the normal distribution of variables. Normally distributed data were assessed for variance using the *F* test. A Student's *t* test was used for comparisons between two unpaired groups with homogeneous variances. Welch's *t* tests were applied for comparisons between two unpaired groups with heterogeneous variances. The Wilcoxon rank‐sum test was used for comparison between two unpaired groups when the data were not normally distributed. Values were considered statistically significant at *P* < 0.05. Statistical analysis was performed using JMP^®^12 (SAS Institute, Cary, NC, USA), and graphs were created using SigmaPlot 14 (Systat Software, San Jose, CA, USA).

## 3 RESULTS

### 3.1 Growth retardation and low BMD

**FIGURE 1:**
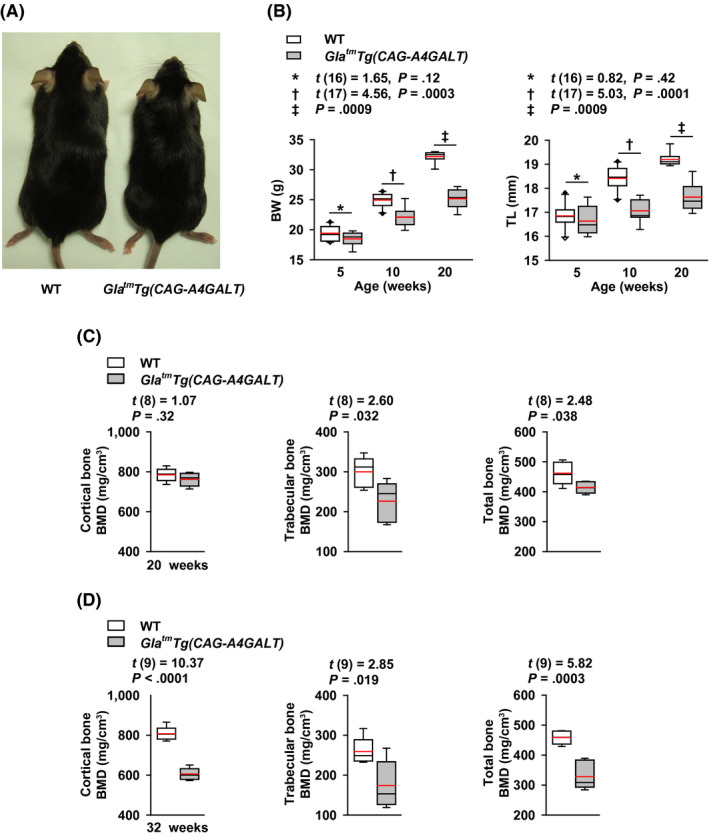
*Gla^tm^Tg(CAG‐A4GALT)* mice failure to thrive and exhibit low bone mineral density (BMD). A, A 20‐wk‐old male WT mouse (left) and *Gla^tm^Tg(CAG‐A4GALT)* mouse (right). B, Body weight (BW) and tibia length (TL) of *Gla^tm^Tg(CAG‐A4GALT)* (5 wk old, n = 8; 10 wk, n = 9; and 20 wk, n = 8) and WT mice (5 wk old, n = 10; 10 wk, n = 10; and 20 wk, n = 8) mice. C, µCT of the BMD of 20‐wk‐old *Gla^tm^Tg(CAG‐A4GALT)* (n = 5) and WT (n = 5) mice. D, µCT of the BMD of 32‐wk‐old *Gla^tm^Tg(CAG‐A4GALT)* (n = 6) and WT (n = 5) mice. In box‐and‐whisker plots (B–D), center lines represent the median, box limits represent quartiles, whiskers represent the 10th and 90th percentiles, black diamonds outside the reach of the whiskers represent minima or maxima, and red lines represent the mean. Differences between groups were evaluated with the Student's *t* test or Wilcoxon rank‐sum test; data are shown as *t* (integral degree of freedom) = *t* value and *P* value (Student's *t* test) or a *P* value only (Wilcoxon rank‐sum test)

Male patients with Fabry disease often show growth retardation.^3^ Similarly, *Gla^tm^Tg(CAG‐A4GALT)* mice exhibited growth retardation when compared with WT mice (Figure 1A). The BW and tibia length of *Gla^tm^Tg(CAG‐A4GALT)* mice were also lower and shorter than that of WT mice (Figure 1B). We next analyzed BMD using μCT and found that the BMD of *Gla^tm^Tg(CAG‐A4GALT)* mice was lower than that of WT mice in the trabecular bone at 20 weeks of age (Figure 1C) and in both the cortical bone and trabecular bones at 32 weeks of age (Figure 1D). Thus, findings indicate that the development of low BMD is initiated in the trabecular bone.

### 3.2 Accelerated bone resorption and osteomalacia

**FIGURE 2:**
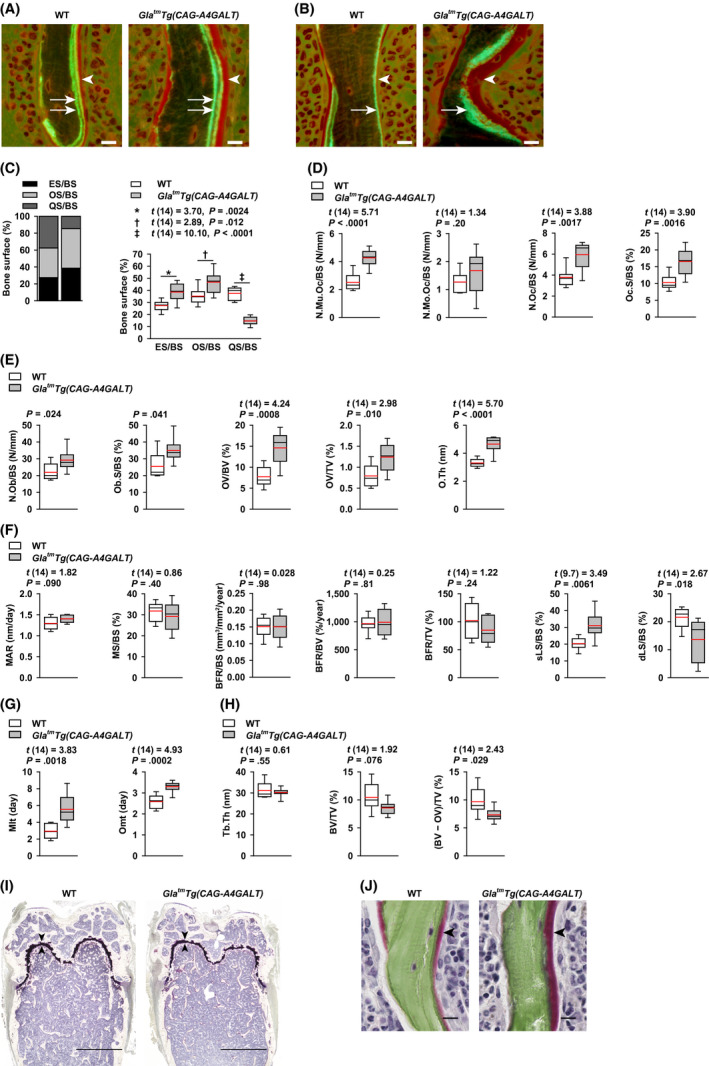
*Gla^tm^Tg(CAG‐A4GALT)* mice exhibit accelerated bone resorption and impaired bone mineralization. Bone histomorphometry of calcein double‐labeled distal femurs in 20‐wk‐old *Gla^tm^Tg(CAG‐A4GALT)* (n = 8) and WT (n = 8) mice (A and B). Representative sections of distal femurs of *Gla^tm^Tg(CAG‐A4GALT)* and WT mice—stained with Villanueva bone stain—under fluorescent light. A, Double‐labeled surface (two arrows) and osteoid thickness (arrowhead). B, Single‐labeled surface (one arrow) and osteoid thickness (arrowhead). Scale bars = 10 µm. C, Stacked bar chart of the bone surface. ES/BS, eroded surface; OS/BS, osteoid surface; QS/BS, quiescent surface. D, Bone‐resorption parameters. N.Mu.Oc/BS, multinuclear osteoclast number; N.Mo.Oc/BS, mononuclear osteoclast number; N.Oc/BS, osteoclast number, including both mononuclear and multinuclear cells; Oc.S/BS, osteoclast surface. E, Bone‐formation parameters (osteoid formation parameters). N.Ob/BS, osteoblast number; Ob.S/BS, osteoblast surface; OV/BV, osteoid volume; OV/TV, osteoid volume; O.Th, osteoid thickness. F, Bone‐formation parameters (bone mineralization parameters). MAR, mineral apposition rate; MS/BS, mineralizing surface; BFR/BS, bone formation rate; BFR/BV, bone formation rate; BFR/TV, bone formation rate; sLS/BS, single‐labeled surface; dLS/BS, double‐labeled surface. G, Parameters of both osteoid formation and bone mineralization. Mlt, mineralization lag time; Omt, osteoid maturation time. H, Bone‐volume parameters. Tb.Th, trabecular thickness; BV/TV, bone volume. Bone volume includes osteoid volume. As there is no standard calcified bone‐volume parameter, we defined it as (BV − OV)/TV, [(bone volume − osteoid volume)/tissue volume]. I, Representative sections of distal femurs of *Gla^tm^Tg(CAG‐A4GALT)* and WT mice (Villanueva bone stain) under natural light. Arrowhead indicates the growth plate. Scale bars = 1 mm. J, Villanueva Goldner bone stained distal femurs of *Gla^tm^Tg(CAG‐A4GALT)* and WT mice under natural light. Osteoid thickness (red, arrowhead) and mineralization tissue (green) are shown. Scale bars = 10 µm. In box‐and‐whisker plots (C–H), center lines represent the median, box limits represent quartiles, whiskers represent the 10th and 90th percentiles, and red lines represent the mean. Differences between groups were evaluated with the Student's *t* test, Welch's *t* test, or Wilcoxon rank‐sum test; data are shown as *t* (integral degree of freedom) = *t* value and *P* value (Student's *t* test); *t* (mixed decimal degree of freedom) = *t* value and *P* value (Welch's *t* test); or a *P* value only (Wilcoxon rank‐sum test)

We performed bone histomorphometric analysis for the bone status in *Gla^tm^Tg(CAG‐A4GALT)* mice (Figure 2A–J). Bone‐surface and resorption parameters revealed accelerated bone resorption and formation (Figure 2C) and increased bone resorption (Figure 2D), respectively. Bone‐formation involves osteoid formation and subsequent mineralization. Investigation of osteoid formation parameters (Figure 2A,B,E) revealed hyperosteoidosis; however, there was no increase in bone‐mineralization parameters (Figure 2F) corresponding to hyperosteoidosis. Increased single‐labeled surface/bone surface and decreased double‐labeled surface/bone surface (dLS/BS) indicated impaired bone mineralization. We made sure that the two sections per sample were the same. Notably, samples completely lacking double labels were absent as shown in dLS/BS (Figure 2F). Moreover, parameters associated with osteoid formation and bone mineralization showed abnormalities (Figure 2G). Although osteoporosis is characterized by normal mineralization lag time (Mlt) and osteoid maturation time (Omt),^10^, ^13^, ^24^ *Gla^tm^Tg(CAG‐A4GALT)* mice displayed longer Mlt and Omt than that in WT mice, ruling out the presence of osteoporosis and supporting a diagnosis of osteomalacia.^10^ Bone‐volume parameters, including osteoid volume, were not altered, except for a decrease in calcified bone‐volume parameter, similar to that observed in case of BMD (Figure 2H). Meanwhile, the growth plates of *Gla^tm^Tg(CAG‐A4GALT)* mice were not different from those of WT mice (Figure 2I). Considering that osteomalacia is characterized by normal growth plates and impaired mineralization of the osteoid, whereas rickets is characterized by impaired mineralization of cartilaginous growth plates and only occurs in children,^26^ the phenotype of the *Gla^tm^Tg(CAG‐A4GALT)* mice is consistent with the osteomalacia‐type phenotype. Additionally, the extent of osteoid in *Gla^tm^Tg(CAG‐A4GALT)* mice was much higher than that of WT mice, as evidenced by Villanueva Goldner staining (Figure 2J).

### 3.3 The levels of plasma bone turnover markers: ALP, P1NP, and CTX‐I

**FIGURE 3:**
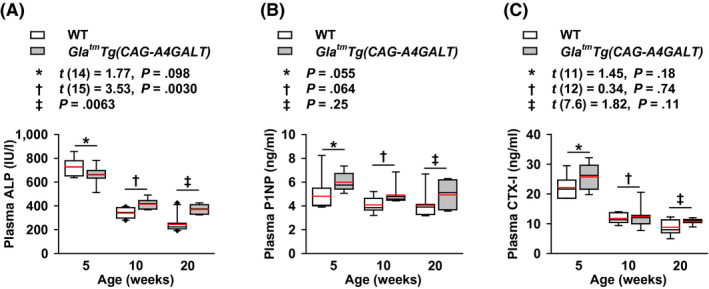
Levels of plasma bone turnover markers: alkaline phosphatase (ALP), procollagen type I N‐terminal propeptide (P1NP), and C‐terminal telopeptide of type I collagen (CTX‐I) in *Gla^tm^Tg(CAG‐A4GALT)* mice. A–C, Plasma ALP (A), P1NP (B), and CTX‐I (C) levels; *Gla^tm^Tg(CAG‐A4GALT)* mice at 5 wk old, n = 8 (ALP), 6 (P1NP), and 7 (CTX‐I); 10 wk, n = 7 (ALP, P1NP, CTX‐I); and 20 wk, n = 7 (ALP, P1NP, CTX‐I). WT mice at 5 wk old, n = 8 (ALP) and 6 (P1NP, CTX‐I); 10 wk, n = 10 (ALP) and 7 (P1NP, CTX‐I); and 20 wk, n = 10 (ALP) and 7 (P1NP, CTX‐I). In box‐and‐whisker plots, center lines represent the median, box limits represent quartiles, whiskers represent the 10th and 90th percentiles, black diamonds outside the reach of the whiskers represent minima or maxima, and red lines represent the mean. Differences between groups were evaluated with the Student's *t* test, Welch's *t* test, or Wilcoxon rank‐sum test; data are shown as *t* (integral degree of freedom) = *t* value and *P* value (Student's *t* test); *t* (mixed decimal degree of freedom) = *t* value and *P* value (Welch's *t* test); or a *P* value only (Wilcoxon rank‐sum test)

Marked liver damage was absent in *Gla^tm^Tg(CAG‐A4GALT)* mice.^17^ Therefore, ALP produced by osteoblasts was used as a marker of bone formation. Plasma ALP levels were not higher in *Gla^tm^Tg(CAG‐A4GALT)* mice than that in WT mice at 5 weeks of age, but became higher starting by 10 weeks (Figure 3A), consistent with the presence of accelerated bone formation and osteomalacia.^27^ Because osteoporosis is not accompanied by osteoblast proliferation, the elevation in plasma ALP levels does not support the hypothesis that osteoporosis is the primary cause of low BMD. We further used P1NP and CTX‐I as markers of bone formation and resorption, respectively^28^, ^29^; although plasma P1NP and CTX‐I levels in *Gla^tm^Tg(CAG‐A4GALT)* and WT mice were not significantly different, they showed a higher trend in *Gla^tm^Tg(CAG‐A4GALT)* mice (Figure 3B,C). These findings complement our histomorphometry results (Figure 2C–F).

### 3.4 Fluctuations in blood Ca^2+^ and plasma P levels and renal P wasting

**FIGURE 4:**
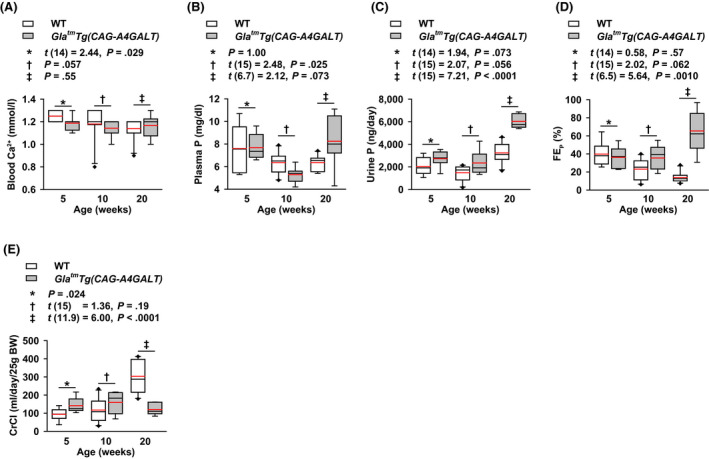
*Gla^tm^Tg(CAG‐A4GALT)* mice exhibit alterations in blood ionized calcium (Ca^2+^), and fluctuations in plasma phosphate (P) levels, and renal P wasting. A, Blood Ca^2+^ levels. B, Plasma P levels. C, Urine excretion of P. D, Fractional excretion of P (FE_P_). E, Creatinine clearance (CrCl). *Gla^tm^Tg(CAG‐A4GALT)* mice at 5 wk old, n = 8; 10 wk, n = 7; and 20 wk, n = 7. WT mice at 5 wk old, n = 8; 10 wk, n = 10; and 20 wk, n = 10 [note that the value of blood Ca^2+^ for one 20‐wk‐old *Gla^tm^Tg(CAG‐A4GALT)* mouse was not obtained due to a technical error]. In box‐and‐whisker plots, center lines represent the median, box limits represent quartiles, whiskers represent the 10th and 90th percentiles, black diamonds outside the reach of the whiskers represent minima or maxima, and red lines represent the mean. Differences between groups were evaluated with the Student's *t* test, Welch's *t* test, or Wilcoxon rank‐sum test; data are shown as *t* (integral degree of freedom) = *t* value and *P* value (Student's *t* test); *t* (mixed decimal degree of freedom) = *t* value and *P* value (Welch's *t* test); or a *P* value only (Wilcoxon rank‐sum test)

In our previous study,^18^ *Gla^tm^Tg(CAG‐A4GALT)* mice displayed renal Ca wasting (hypercalciuria) and elevated plasma Ca levels (hypercalcemia) by 20 weeks of age, indicating that hypercalcemia causes hypercalciuria. In the present study, blood Ca^2+^ was lower in *Gla^tm^Tg(CAG‐A4GALT)* mice than that in WT mice by 5 weeks of age (Figure 4A), although this difference between strains disappeared by 20 weeks. This suggests the presence of factors that reduce and then restore blood Ca^2+^ levels.

Compared with WT mice, plasma P levels in *Gla^tm^Tg(CAG‐A4GALT)* mice fluctuated around normophosphatemia at 5 weeks of age, hypophosphatemia at 10 weeks (Figure 4B), and hyperphosphatemia at 20 weeks, indicating the presence of factors that induce such fluctuations. Urine excretion of P tended to be higher in *Gla^tm^Tg(CAG‐A4GALT)* mice than that in WT mice by 5 weeks of age and progressively increased by 20 weeks (Figure 4C). FE_P_ tended to be higher in *Gla^tm^Tg(CAG‐A4GALT)* mice than that in WT mice by 10 weeks of age (Figure 4D). Unlike the impaired PT in chronic kidney disease (CKD), the unimpaired PT maintains the renal P excretion system. Thus, *Gla^tm^Tg(CAG‐A4GALT)* mice display renal P wasting (hyperphosphaturia) by 20 weeks of age, which is an important causal factor of osteomalacia. Considering the time courses of blood Ca^2+^ and plasma P levels, we suspect the possible involvement of PTH in mediating these fluctuations.

### 3.5 Reduction in CrCl

We had previously reported that *Gla^tm^Tg(CAG‐A4GALT)* mice show renal dysfunction, as evidenced by plasma Cr and blood urea nitrogen levels at 10 weeks of age.^18^ Similarly, the CrCl test of *Gla^tm^Tg(CAG‐A4GALT)* mice did not show the progressive increase observed in WT mice, and was 40% of the WT level at 20 weeks of age (Figure 4E). It is likely that the fibrosis and inflammation around impaired TALs—described in our previous report^18^—decreased the CrCl.

### 3.6 Increase in plasma PTH levels preceded the increase in plasma FGF‐23 levels

**FIGURE 5:**
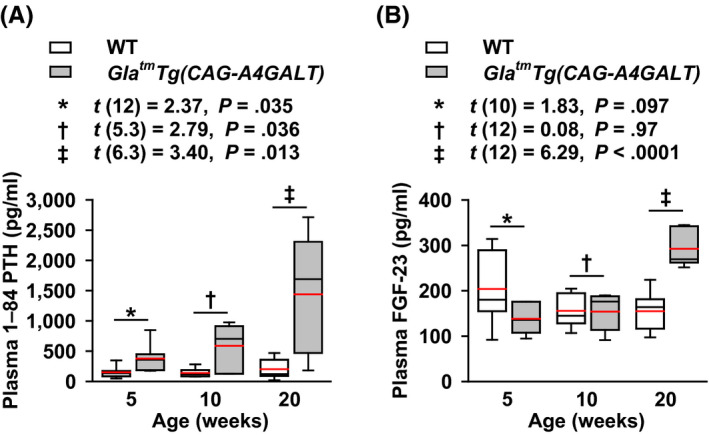
*Gla^tm^Tg(CAG‐A4GALT)* mice exhibit alterations in plasma 1–84 parathyroid hormone (PTH) and fibroblast growth factor 23 (FGF‐23) levels. A and B, Plasma PTH (A) and FGF‐23 (B) levels; *Gla^tm^Tg(CAG‐A4GALT)* mice at 5 wk old, n = 7 (PTH) and 6 (FGF‐23); 10 wk, n = 6 (PTH) and 7 (FGF‐23); and 20 wk, n = 7 (PTH, FGF‐23). WT mice at 5 wk old, n = 7 (PTH) and 6 (FGF‐23); 10 wk, n = 7 (PTH, FGF‐23); and 20 wk, n = 7 (PTH, FGF‐23). In box‐and‐whisker plots, center lines represent the median, box limits represent quartiles, whiskers represent the 10th and 90th percentiles, and red lines represent the mean. Differences between groups were evaluated with the Student's *t* test or Welch's *t* test; data are shown as *t* (integral degree of freedom) = *t* value and *P* value (Student's *t* test) or *t* (mixed decimal degree of freedom) = *t* value and *P* value (Welch's *t* test)

Plasma 1–84 PTH levels were higher in *Gla^tm^Tg(CAG‐A4GALT)* mice than that in WT mice by 5 weeks of age as a compensatory response to low blood Ca^2+^. This progressed to secondary hyperparathyroidism by 20 weeks of age (Figure 5A), which caused both hypercalcemia and hyperphosphatemia due to accelerated bone resorption by PTH. Moreover, hypophosphatemia at 10 weeks of age can be explained by enhanced phosphaturic action due to elevated plasma PTH levels. Thus, PTH is associated with the onset and progression of low BMD due to accelerated bone resorption and osteomalacia.

FGF‐23 regulates P metabolism.^30^ Compared with PTH, FGF‐23 is a sensitive early biomarker of dysregulated P metabolism in patients with CKD and normal serum P levels.^31^ Differences in plasma FGF‐23 levels between strains were not detected by 10 weeks of age (Figure 5B), indicating that FGF‐23 is not associated with the onset of hyperphosphaturia (Figure 4C). Plasma FGF‐23 levels in *Gla^tm^Tg(CAG‐A4GALT)* mice increased in response to hyperphosphatemia due to secondary hyperparathyroidism by 20 weeks of age. Thus, FGF‐23 was determined to be an exacerbating factor of hyperphosphaturia *via* phosphaturic action.

### 3.7 Expression of SLC34A1 and megalin in the PT

**FIGURE 6:**
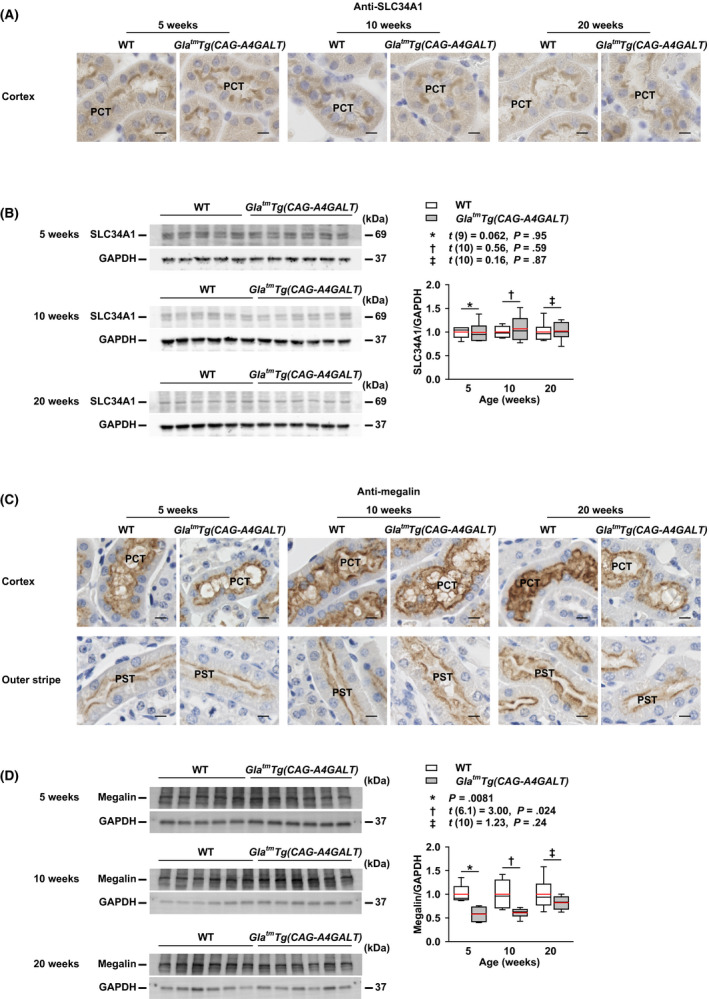
*Gla^tm^Tg(CAG‐A4GALT)* mice exhibit normal expression of solute carrier family 34 member 1 (SLC34A1) and decreased expression of megalin in the proximal tubule (PT). A, Micrographs of SLC34A1 expression in the proximal convoluted tubule (PCT) of *Gla^tm^Tg(CAG‐A4GALT)* and WT mice (n = 3/group). B, Representative (n = 2) western blots showing SLC34A1 expression in *Gla^tm^Tg(CAG‐A4GALT)* (5 wk old, n = 6; 10 wk, n = 6; and 20 wk, n = 6) and WT (5 wk old, n = 5; 10 wk, n = 6; and 20 wk, n = 6) mice. C, Micrographs of megalin expression in the PCT and proximal straight tubule (PST) of *Gla^tm^Tg(CAG‐A4GALT)* and WT mice (n = 3/group). D, Representative western blots showing megalin expression in the same mice as in panel B. In box‐and‐whisker plots (B and D), center lines represent the median, box limits represent quartiles, whiskers represent the 10th and 90th percentiles, and red lines represent the mean. Differences between groups were evaluated with the Student's *t* test, Welch's *t* test, or Wilcoxon rank‐sum test; data are shown as *t* (integral degree of freedom) = *t* value and *P* value (Student's *t* test); *t* (mixed decimal degree of freedom) = *t* value and *P* value (Welch's *t* test); or a *P* value only (Wilcoxon rank‐sum test). Scale bars = 10 μm

SLC34A1, also known as an NaPi‐IIa cotransporter, is localized at the brush border membrane of PT cells and is responsible for reabsorbing more than 70% of the filtered P in the PT.^32^, ^33^ In *Gla^tm^Tg(CAG‐A4GALT)* mice, SLC34A1 was exclusively localized at the brush border membrane of proximal convoluted tubule (PCT) cells, in a manner similar to that in WT mice (Figure 6A). The protein levels of SLC34A1 were also similar in both strains (Figure 6B). Normal PCT morphology excluded the possibility of reduced SLC34A1 expression in the PCT.

Megalin is localized at the brush border membrane of PT cells and mediates the internalization of SLC34A1, which is required for PTH to suppress P reabsorption.^34^ It is also involved in P metabolism as megalin is the receptor for PTH endocytosis.^35^ In *Gla^tm^Tg(CAG‐A4GALT)* mice, megalin expression at the brush border membrane of PT cells was slightly lower than that in WT mice (Figure 6C); megalin protein levels were also lower at 5 and 10 weeks of age (Figure 6D).

### 3.8 Alterations in vitamin D activating enzyme CYP27B1 and inactivating enzyme CYP24A1 levels in the kidney

**FIGURE 7:**
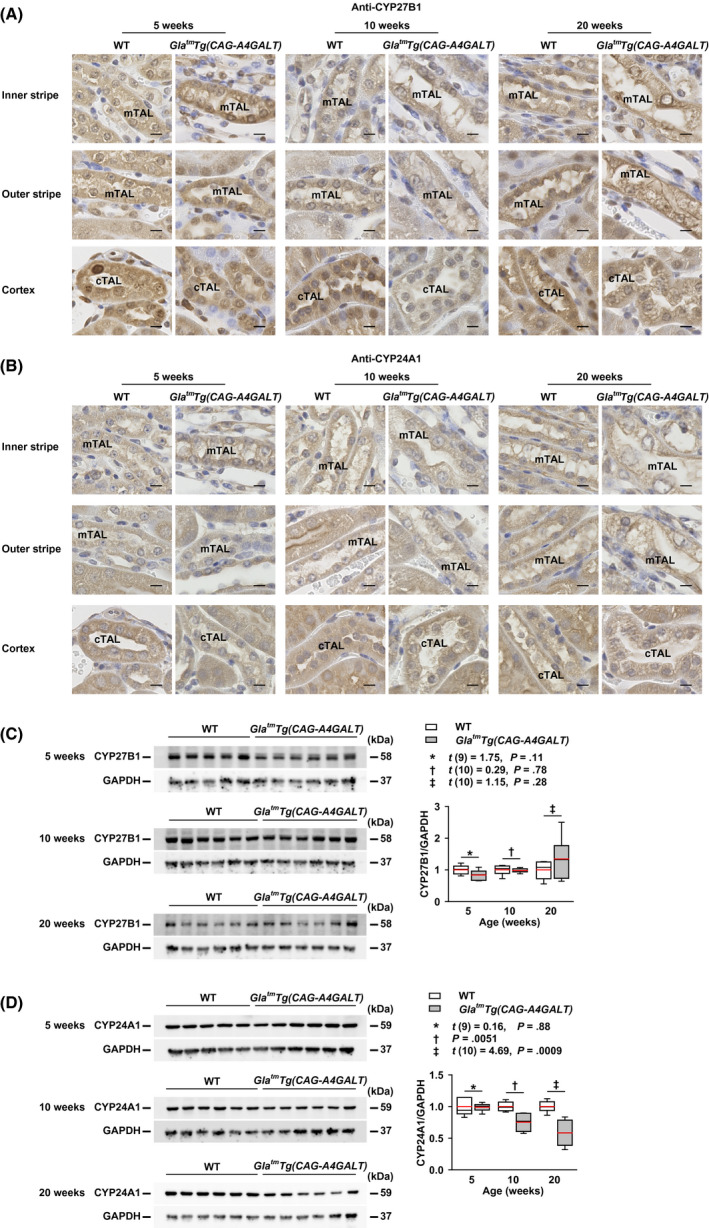
*Gla^tm^Tg(CAG‐A4GALT)* mice exhibit alterations in 1α‐hydroxylase (CYP27B1) and 24‐hydroxylase (CYP24A1) levels. A and B, Micrographs of CYP27B1 (A) and CYP24A1 (B) expression in the thick ascending limb (TAL) of *Gla^tm^Tg(CAG‐A4GALT)* and WT mice (n = 3/group). Scale bars = 10 μm. C and D, Representative (out of two experiments) western blots showing CYP27B1 (C) and CYP24A1 (D) expression in the same mice as in Figure 6B. In box‐and‐whisker plots (C and D), center lines represent the median, box limits represent quartiles, whiskers represent the 10th and 90th percentiles, and red lines represent the mean. Differences between groups were evaluated with the Student's *t* test or Wilcoxon rank‐sum test; data are shown as *t* (integral degree of freedom) = *t* value and *P* value (Student's *t* test) or a *P* value only (Wilcoxon rank‐sum test). cTAL, cortical thick ascending limb; mTAL, medullary thick ascending limb

CYP27B1 (Figure 7A, Figure S2A) and CYP24A1 (Figure 7B, Figure S3A) were found expressed in the glomeruli, PTs, TALs, DCTs, and CDs, as previously reported.^20^, ^21^, ^36^, ^37^, ^38^ In *Gla^tm^Tg(CAG‐A4GALT)* mice, the staining intensity of CYP27B1 and CYP24A1 progressively decreased in impaired tubular segments. mTALs showed the most severely reduced staining intensity, followed by the cortical thick ascending limbs, DCTs, and CDs. CYP27B1 staining intensity in podocytes was similar between the two strains (Figure S2B), whereas CYP24A1 staining intensity in podocytes progressively decreased in *Gla^tm^Tg(CAG‐A4GALT)* mice (Figure S3B). Staining intensity of the unimpaired PT did not show a compensatory response to the reduction in CYP27B1 and CYP24A1 expression in other tubular segments. Unlike CYP27B1 levels in the tubular segments, CYP27B1 protein levels in the whole kidney were similar between the two strains (Figure 7C), indicating that CYP27B1 is also produced at sites other than nephrons, *ie*, the glomerulus and tubule, in *Gla^tm^Tg(CAG‐A4GALT)* mice. Meanwhile, CYP24A1 protein levels in the whole kidney were significantly lower in *Gla^tm^Tg(CAG‐A4GALT)* mice than those in WT mice by 10 weeks of age. Elevated plasma PTH levels were also found to contribute to the reduction in CYP24A1 protein levels (Figure 7D).

### 3.9 CYP27B1 production by infiltrating interstitial mononuclear cells

**FIGURE 8:**
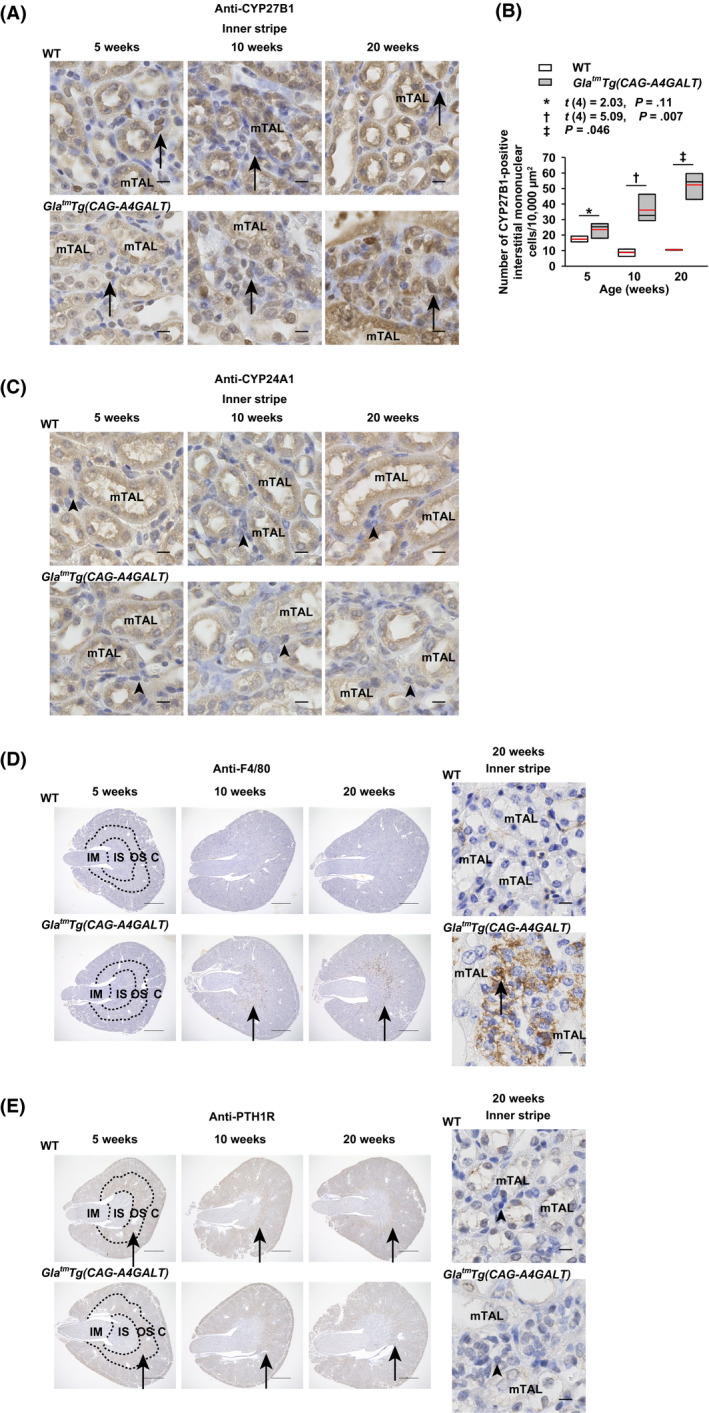
The infiltrating interstitial mononuclear cells of *Gla^tm^Tg(CAG‐A4GALT)* mice produce 1α‐hydroxylase (CYP27B1). A, Micrographs of CYP27B1‐positive mononuclear cells in the inner stripe of *Gla^tm^Tg(CAG‐A4GALT)* and WT mice (n = 3/group) mouse kidneys. Arrows indicate CYP27B1‐positive mononuclear cells. Scale bars = 10 μm. B, Quantification of the CYP27B1‐positive mononuclear cells in the inner stripe of *Gla^tm^Tg(CAG‐A4GALT)* and WT mice (n = 3/group) mouse kidneys. CYP27B1‐positive mononuclear cells in three 10000‐μm^2^ square fields with noticeable mononuclear cells were counted, and the mean value is shown for each mouse. C, Micrographs of CYP24A1‐negative mononuclear cells in the inner stripe of *Gla^tm^Tg(CAG‐A4GALT)* and WT mice (n = 3/group) mouse kidneys. Arrowheads indicate CYP24A1‐negative mononuclear cells. Scale bars = 10 μm. D, Micrographs of F4/80‐positive macrophages in the inner stripe of *Gla^tm^Tg(CAG‐A4GALT)* and WT mice (n = 3/group) mouse kidneys. Arrows indicate F4/80‐positive macrophages. Scale bars = 1 mm (left) and 10 μm (right). E, Micrographs of PTH1R‐negative mononuclear cells in the inner stripe of *Gla^tm^Tg(CAG‐A4GALT)* and WT mice (n = 3/group) mouse kidneys. Arrows indicate PTH1R‐positive PST. Scale bars = 1 mm (left) and 10 μm (right). In box‐and‐whisker plots (B), center lines represent the median, box limits represent quartiles, whiskers represent the 10th and 90th percentiles, and red lines represent the mean. Differences between groups were evaluated with the Student's *t* test or Wilcoxon rank‐sum test; data are shown as *t* (integral degree of freedom) = *t* value and *P* value (Student's *t* test) or a *P* value only (Wilcoxon rank‐sum test). C, cortex; IM, inner medulla; IS, inner stripe; mTAL, medullary thick ascending limb; OS, outer stripe; PST, proximal straight tubule; PTH1R, parathyroid hormone 1 receptor

Infiltrating mononuclear cells, *ie*, lymphocytes, monocytes, and macrophages, around impaired TALs in *Gla^tm^Tg(CAG‐A4GALT)* mice progressively increased in the inner stripe of kidneys, and were CYP27B1‐positive (Figure 8A,B) and CYP24A1‐negative (Figure 8C). Some were positive for the macrophage‐specific antigen F4/80‐positive (Figure 8D). Interstitial mononuclear cells with similar characteristics were also present in WT mice in the inner stripe. Thus, the site of CYP27B1 production, other than nephrons corresponded to the presence of infiltrating mononuclear cells. To investigate the effect of PTH on CYP27B1 production in these cells, we examined the PTH1R expression and found that it was predominantly expressed in proximal straight tubule (PST) cells in the outer stripe in both strains (Figure 8E). CYP27B1‐positive mononuclear cells were PTH1R‐negative (Figure 8E), indicating that PTH does not stimulate CYP27B1 production in these cells.

### 3.10 Increased levels of plasma vitamin D

**FIGURE 9:**
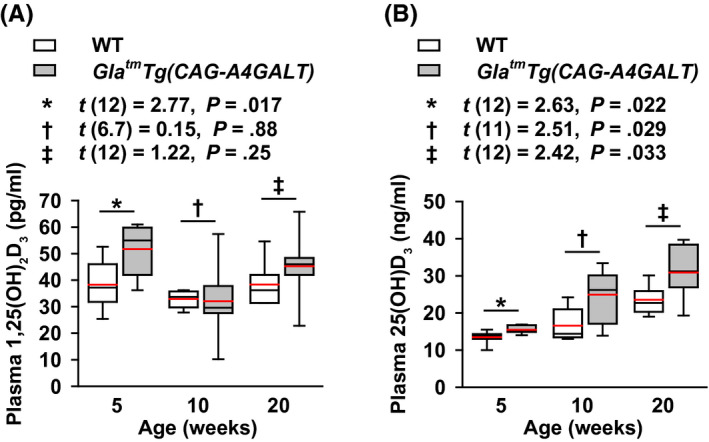
*Gla^tm^Tg(CAG‐A4GALT)* mice exhibit an increase in vitamin D. A and B, Plasma 1,25(OH)_2_D_3_ (A) and 25(OH)D_3_ (B) levels in *Gla^tm^Tg(CAG‐A4GALT)* (5 wk old, n = 7; 10 wk, n = 7; and 20 wk, n = 7) and WT (5 wk old, n = 7; 10 wk, n = 6; and 20 wk, n = 7) mice. In box‐and‐whisker plots, center lines represent the median, box limits represent quartiles, whiskers represent the 10th and 90th percentiles, and red lines represent the mean. Differences between groups were evaluated with the Student's *t* test or Welch's *t* test; data are shown as *t* (integral degree of freedom) = *t* value and *P* value (Student's *t* test) or *t* (mixed decimal degree of freedom) = *t* value and *P* value (Welch's *t* test). 25(OH)D_3_, 25‐hydroxyvitamin D_3_; 1,25(OH)_2_D_3_, 1α,25‐dihydroxyvitamin D_3_

Plasma 1,25(OH)_2_D_3_ levels were higher in *Gla^tm^Tg(CAG‐A4GALT)* mice than those in WT mice at 5 weeks of age in response to reduced blood Ca^2+^ or elevated plasma PTH levels (Figure 9A). Thereafter, plasma 1,25(OH)_2_D_3_ levels were not significantly different between the strains, but tended to be higher in *Gla^tm^Tg(CAG‐A4GALT)* mice in response to reduced blood Ca^2+^ or elevated plasma PTH levels (Figure 9A). The lack of a significant increase in the 1,25(OH)_2_D_3_ production in response to elevated plasma PTH is possibly because CYP27B1 expression in *Gla^tm^Tg(CAG‐A4GALT)* mice was not different from that in WT mice with normal plasma PTH levels. Plasma 25(OH)D_3_—the best measure of vitamin D status^39^—levels was higher in *Gla^tm^Tg(CAG‐A4GALT)* mice than that in WT mice by 5 weeks of age (Figure 9B). High plasma 25(OH)D_3_ levels indicate impaired inactivation of 25(OH)D_3_ due to reduced expression of CYP24A1. Overall, the results indicate that *Gla^tm^Tg(CAG‐A4GALT)* mice do not have a vitamin D deficiency, which is a causal factor of osteomalacia (Figure 2A).

## 4 DISCUSSION

Using the *Gla^tm^Tg(CAG‐A4GALT)* Fabry mouse model with impaired mTAL, we demonstrated that mTAL dysfunction, *ie*, a decrease in blood Ca^2+^, induces an elevation in the levels of phosphaturic hormone PTH and that, ultimately, secondary hyperparathyroidism results in accelerated bone resorption and subsequent hypercalcemia^18^ and hyperphosphatemia, followed by hypercalciuria^18^ and hyperphosphaturia, thereby causing osteomalacia. Low BMD resulted from accelerated bone resorption and osteomalacia, which is the typically suggested mechanism in patients with Fabry disease.

Previous studies that utilized DEXA alone diagnosed the high incidence of low BMD as osteoporosis and osteopenia in patients with Fabry disease.^5^, ^6^, ^7^, ^8^ However, *Gla^tm^Tg(CAG‐A4GALT)* mice also exhibit low BMD, which appears to result from accelerated bone resorption and osteomalacia. Thus, densitometric analyses may misinterpret the low BMD caused by osteomalacia as osteoporosis.^40^ Bone histomorphometry—the only method able to diagnose osteomalacia^13^—may reveal that osteomalacia as well as osteoporosis are responsible for the low BMD in patients with Fabry disease.

Osteopenia is present in approximately 50% of the patients with untreated Fabry disease,^6^ while 20% of the patients with Fabry disease have hyperparathyroidism (secondary hyperparathyroidism caused by CKD).^6^ In CKD, a vitamin D deficiency associated with elevated FGF‐23 levels is thought to represent the initial event in the development of secondary hyperparathyroidism.^41^ In the current model of mTAL‐impaired *Gla^tm^Tg(CAG‐A4GALT)* mice with normal CrCl, a decrease in blood Ca^2+^ led to an elevation in plasma PTH levels and subsequent secondary hyperparathyroidism. However, the elevation in plasma FGF‐23 levels was not associated with the onset of increased plasma PTH levels. Unlike in *Gla^tm^Tg(CAG‐A4GALT)* mice, both glomerular injury and tubular injury—representing mTAL dysfunction—occur in patients with Fabry nephropathy. Therefore, an increase in plasma FGF‐23 levels as well as a decrease in blood Ca^2+^ may be responsible for the onset of secondary hyperparathyroidism and may be considered the cause of low BMD in patients with Fabry nephropathy.

CYP24A1 and CYP27B1 expression in the tubules is elevated in CKD patients with acute renal inflammation, *ie*, renal vasculitis or interstitial nephritis,^42^ and in adenine‐treated uremic rats with marked interstitial fibrosis, severe tubular dilatation, microcystic change, and foci of tubular atrophy.^37^ In contrast, in *Gla^tm^Tg(CAG‐A4GALT)* mice, the expression of CYP24A1 and CYP27B1 was downregulated in tubules with Gb3 accumulation. Thus, the production of these enzymes in impaired tubules may depend on the type of pathological tubular damage.

CYP24A1 plays a critical role in the catabolism of 1,25(OH)_2_D_3_ and 25(OH)D_3_. PTH downregulates CYP24A1 expression,^43^ whereas FGF‐23 upregulates CYP24A1 expression.^44^ Therefore, CYP24A1 expression levels may depend on which is predominant, PTH or FGF‐23. FGF‐23 is elevated before PTH and P,^31^ and CYP24A1 expression is increased in CKD patients, resulting in decreased vitamin D status.^45^ In contrast, the downregulation of CYP24A1 in *Gla^tm^Tg(CAG‐A4GALT)* mice may be attributed to the maintenance or increase in plasma 1,25(OH)_2_D_3_ and 25(OH)D_3_ levels.

The predominant sites of expression of *Cyp27b1* mRNA and CYP27B1 protein are TALs, DCTs, and CDs—under conditions of vitamin D sufficiency—and the PCT under vitamin D deficiency.^20^ In the present study, CYP27B1 expression in the PT of *Gla^tm^Tg(CAG‐A4GALT)* mice did not increase without a corresponding decrease in its expression in the impaired TAL, even though the PT was histologically normal. Thus, in the background of normal or high plasma 1,25(OH)_2_D_3_ and 25(OH)D_3_ levels in *Gla^tm^Tg(CAG‐A4GALT)* mice, the stimulus for the PT to increase CYP27B1 expression may not occur.

Patients with acute renal inflammation exhibit significantly high expression of C‐C motif chemokine ligand (CCL) 2 expression, macrophage infiltration, and macrophage CYP27B1 expression.^42^ The transcript levels of *Ccl2* are upregulated in *Gla^tm^Tg(CAG‐A4GALT)* mice^18^ and murine CD8^+^ T cells also express CYP27B1.^46^ Similarly, infiltrating mononuclear cells around mTALs in *Gla^tm^Tg(CAG‐A4GALT)* mice produced CYP27B1. Considering the presence of CYP27B1‐positive cells even in WT mice, interstitial mononuclear cells may possess the ability to produce CYP27B1 before the onset of inflammation. Moreover, infiltrating CYP27B1‐positive mononuclear cells were CYP24A1‐negative in *Gla^tm^Tg(CAG‐A4GALT)* mice. This contributes to the increased efficiency of 1,25(OH)_2_D_3_ in infiltrating interstitial mononuclear cells compared with that in tubular cells.^47^ Meanwhile, PTH1R‐negative interstitial mononuclear cells in the inner stripe of *Gla^tm^Tg(CAG‐A4GALT)* kidneys indicated that increased PTH levels do not stimulate the production of CYP27B1. Thus, the factor that stimulates CYP27B1 production in interstitial mononuclear cells remains unknown.

SLC34A1 expression in *Gla^tm^Tg(CAG‐A4GALT)* mice was upregulated by P wasting and downregulated by increased levels of the phosphaturic hormones, PTH and FGF‐23; however, the levels were comparable to those in WT mice without P wasting. The SLC34A1 levels were insufficient to reabsorb the excessively filtered P. Although the factors that determine the expression levels of megalin for P reabsorption are unknown, the reduction in megalin expression in *Gla^tm^Tg(CAG‐A4GALT)* mice may indirectly reduce the loss of P *via* reduced internalization of SLC34A1.^34^

Osteomalacia occurs when the existing bone is replaced by unmineralized bone matrix (osteoid) during remodeling in children and adults, or when newly formed bone is not mineralized in time during modeling in children. Rickets occurs when hypomineralization affects the epiphyseal growth plate chondrocytes and adjacent bone metaphysis in growing children.^48^ The growth plate becomes thick, wide, and irregular.^26^ Hence, osteomalacia coexists with rickets in growing children.^48^ If 5‐week‐old *Gla^tm^Tg(CAG‐A4GALT)* mice expressed the factor responsible for causing osteomalacia, then they would exhibit rickets as well as osteomalacia. Therefore, osteomalacia in *Gla^tm^Tg(CAG‐A4GALT)* mice may not be observed at 5 weeks of age but at 20 weeks.

There are several limitations associated with the study. First, the mechanism underlying low BMD may differ between patients with Fabry disease and *Gla^tm^Tg(CAG‐A4GALT)* mice. The present study therefore postulates that osteomalacia may be an alternative cause of low BMD in patients with Fabry disease. Second, although we evaluated Ca^2+^, P, and vitamin D metabolism in the kidney, we did not examine their metabolism in the intestine, which may be involved in the pathophysiology of bone disorders. Nonetheless, we clarified the mechanism underlying the bone disorder in *Gla^tm^Tg(CAG‐A4GALT)* mice. Third, although we examined the mechanism of low BMD from the standpoint of Fabry nephropathy, we did not examine Gb3 accumulation in the bone, as we were unable to prepare bone sections for examining Gb3 accumulation. Therefore, at this stage, we cannot possibly hypothesize whether Gb3 accumulation in the bone is involved in the pathophysiology of this disorder.

**FIGURE 10:**
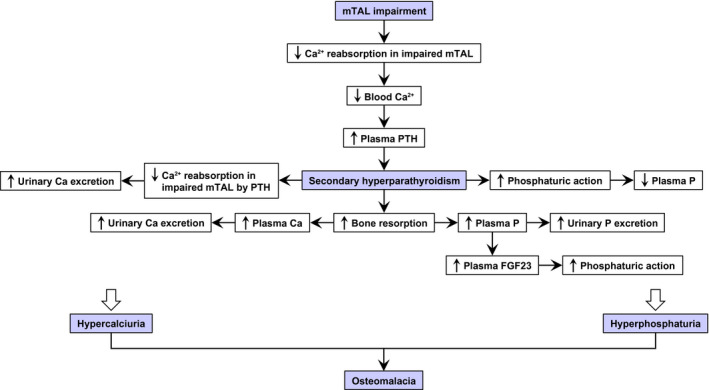
Mechanism underlying the changes in calcium (Ca) and phosphate (P) homeostasis over time in *Gla^tm^Tg(CAG‐A4GALT)* mice with osteomalacia due to secondary hyperparathyroidism. *Gla^tm^Tg(CAG‐A4GALT)* mice were characterized by progressive medullary thick ascending limb (mTAL) impairment without remarkable damage to the glomeruli or proximal tubule (PTs).^18^ Generally, 20%–25% of filtered Ca^2+^ is reabsorbed in the TAL, while very low levels of filtered P are reabsorbed.^30^ Parathyroid hormone (PTH) increases phosphaturic action in PTs, which is evidenced by a decrease in P reabsorption and increase in P excretion. PTH increases reabsorption of Ca^2+^ in the TAL,^30^ decreasing Ca excretion. In *Gla^tm^Tg(CAG‐A4GALT)* mice, mTAL impairment leads to insufficient Ca^2+^ reabsorption.^18^ Low blood Ca^2+^ (Figure 4A) elevated plasma PTH levels by 5 wk of age (Figure 5A), showing progressively increased phosphaturic action, *ie*, decreased P reabsorption in the unimpaired PT and increased urinary P excretion (Figure 4C), and decreased plasma P levels at 10 wk of age (Figure 4B). Secondary hyperparathyroidism developed by 20 wk of age (Figure 5A) and increased bone resorption (Figure 2D), leading to hyperphosphatemia (Figure 4B), which increases urinary P excretion (Figure 4C) and plasma FGF‐23 levels (Figure 5B) with phosphaturic action. Despite the increasing plasma PTH levels (Figure 5A), the progressively impaired mTAL exhibited insufficient urinary Ca reabsorption, leading to increased Ca excretion over time.^18^ Bone resorption (Figure 2D) caused hypercalcemia,^18^ which increased urinary Ca excretion.^18^ Thus, hyperphosphaturia and hypercalciuria result in osteomalacia in *Gla^tm^Tg(CAG‐A4GALT)* mice. FGF‐23, fibroblast growth factor 23

Despite these limitations, we found that the cause of low BMD accelerated bone resorption and osteomalacia in our mouse model of Fabry disease, and that mTAL dysfunction—*ie*, Ca^2+^ loss—primarily caused secondary hyperparathyroidism, leading to bone changes. Based on our findings, we summarized the mechanisms underlying the change in Ca and P homeostasis in *Gla^tm^Tg(CAG‐A4GALT)* mice over time (Figure 10). Our results highlight the importance of mTAL dysfunction in the pathophysiology of Fabry bone disease and indicate that bone histomorphometry should be required for examining the cause of the low BMD observed in patients with Fabry disease. Additional studies are required to verify whether our findings are relevant to human disease and whether they can provide a basis for the development of adequate therapies.

## AUTHOR CONTRIBUTIONS

H. Maruyama and S. Ishii designed the study; H. Maruyama, A. Taguchi, M. Mikame, and S. Ishii performed experiments and analyzed data; H. Lu, N. Tada, M. Ishijima, H. Kaneko, M. Kawai, S. Goto, A. Saito, R. Ohashi, and Y. Nishikawa analyzed data; and H. Maruyama, A. Taguchi, and S. Ishii wrote the paper. All authors contributed to the drafting of the paper and agreed to its contents.

## Supporting information

Fig S1‐S3

Table S1
