# Supplementary figures and images for: Low bone mineral density due to secondary hyperparathyroidism in the GlatmTg(CAG‐A4GALT) mouse model of Fabry disease
Source: FASEB Bioadv. 2020 Jun 10;2(6):365–81. doi: 10.1096/fba.2019-00080 (PMC7325589; doi:10.1096/fba.2019-00080)

Supplemental Figure 1

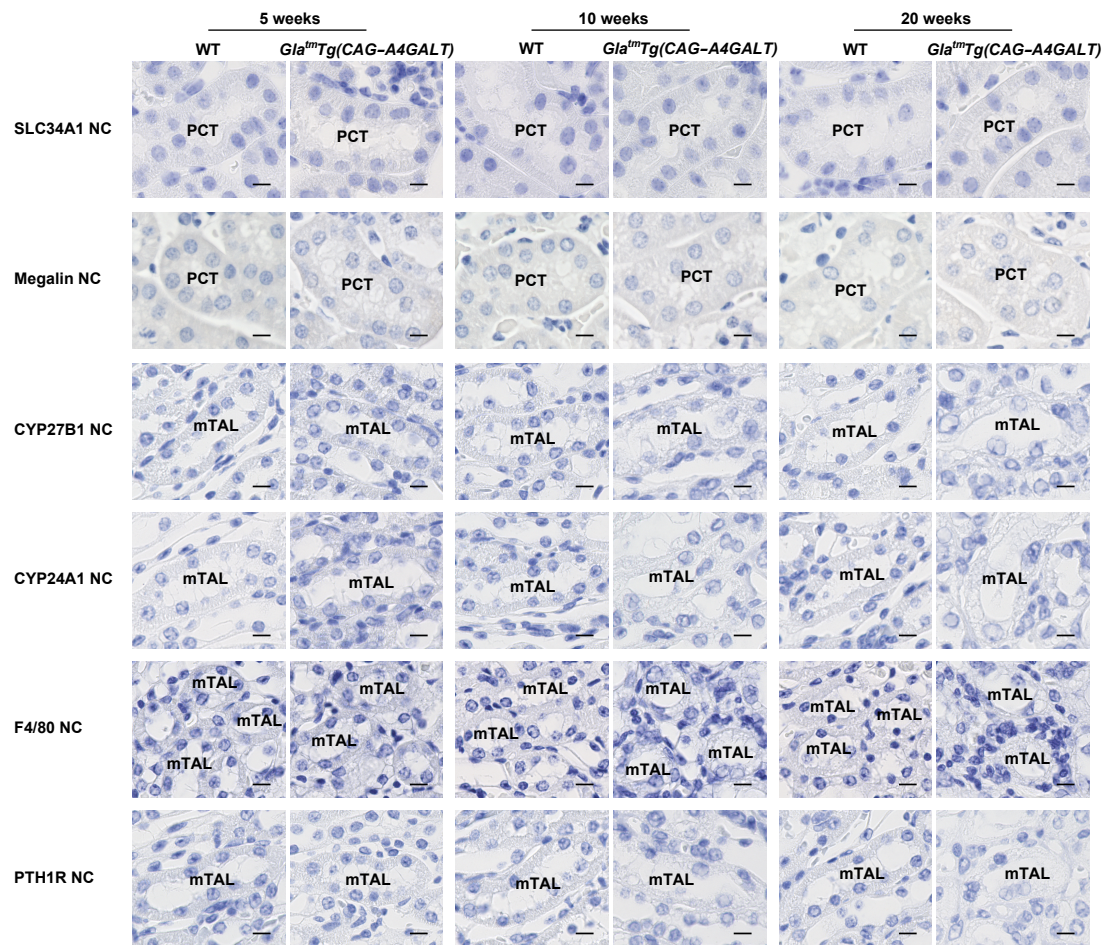

Supplemental Figure 2

(A)

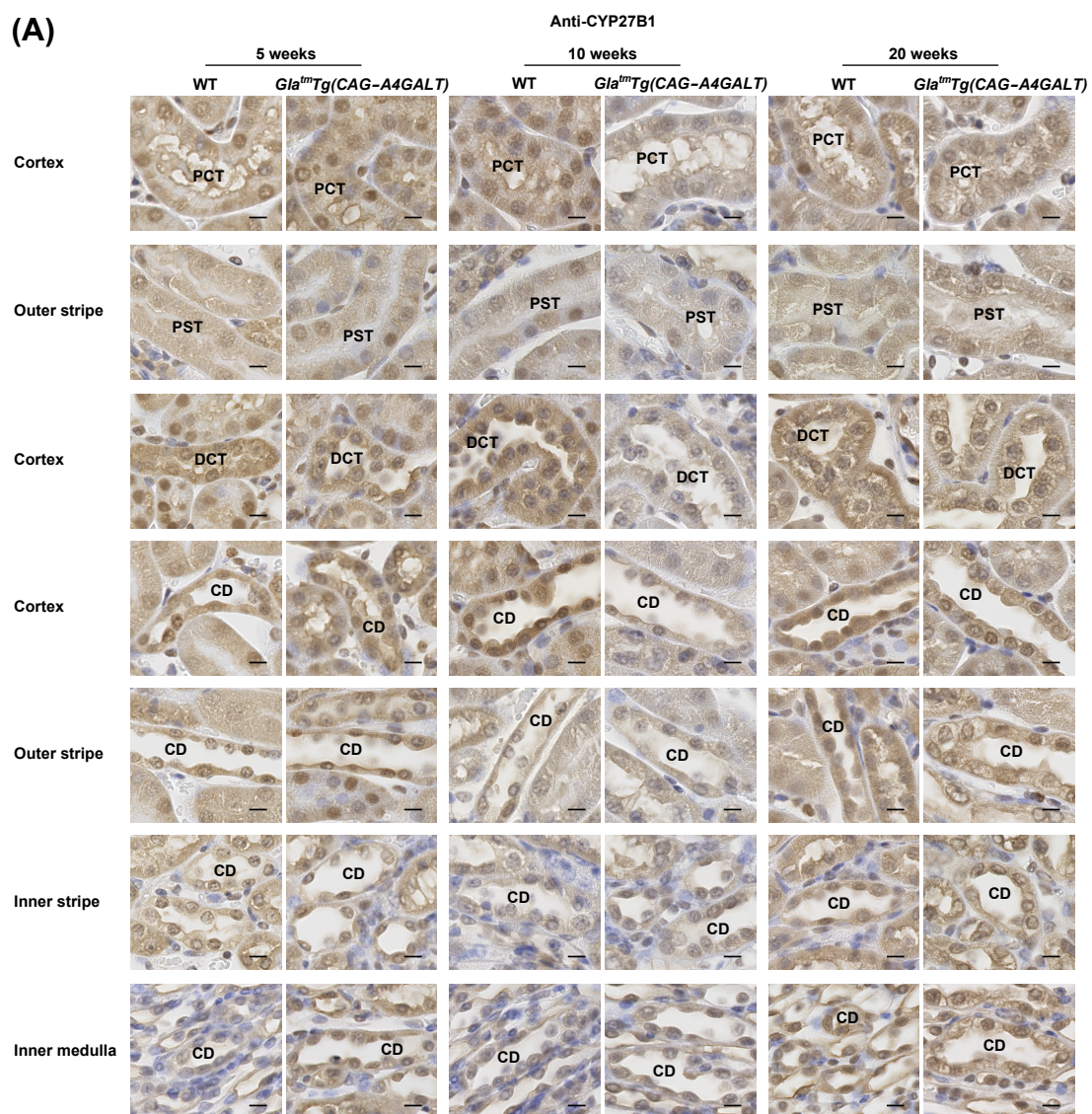

(B)

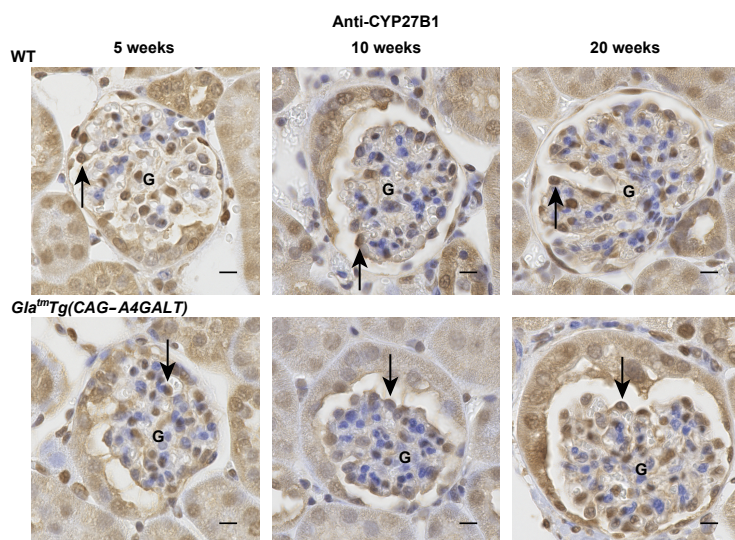

Supplemental Figure 3

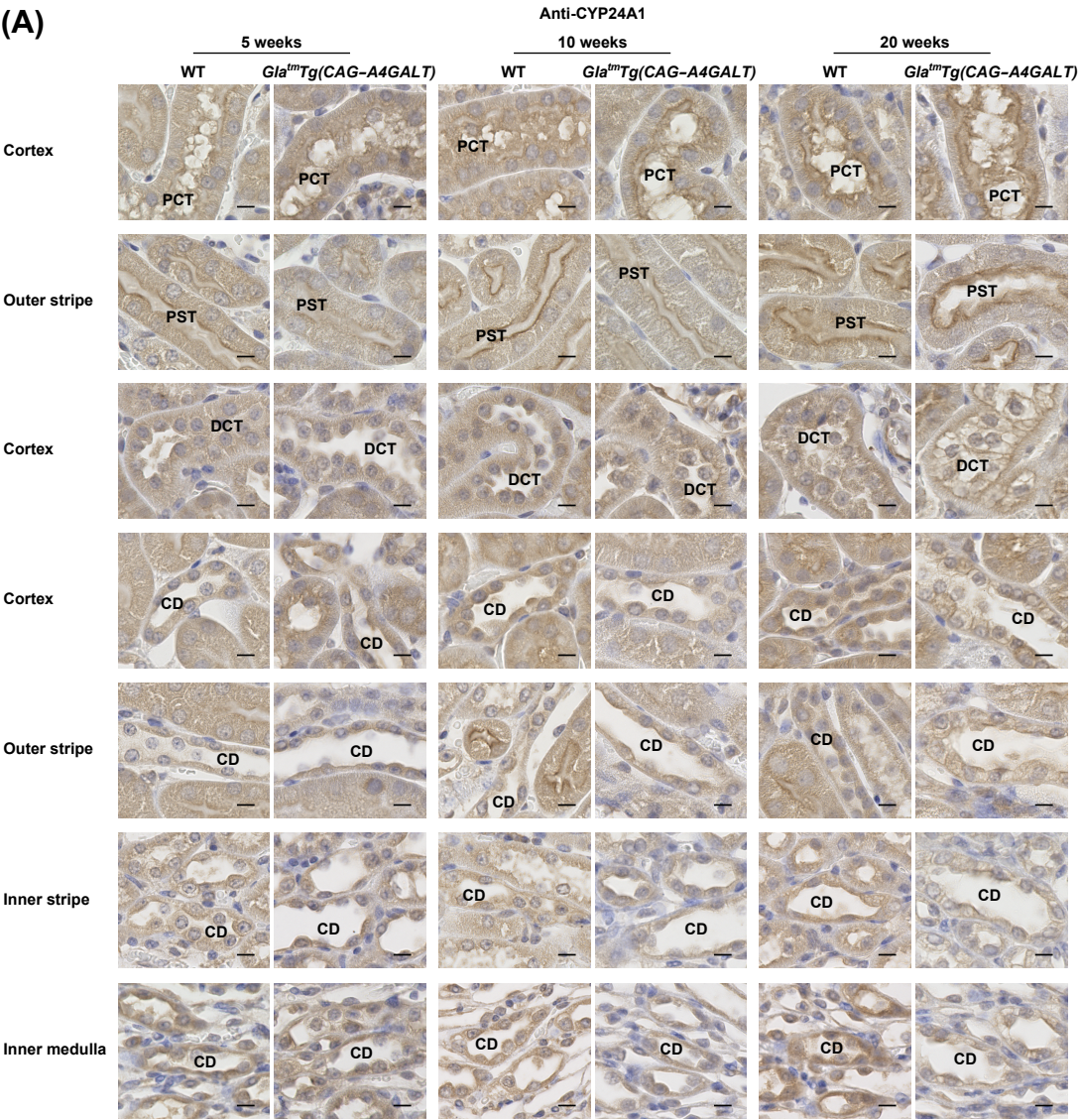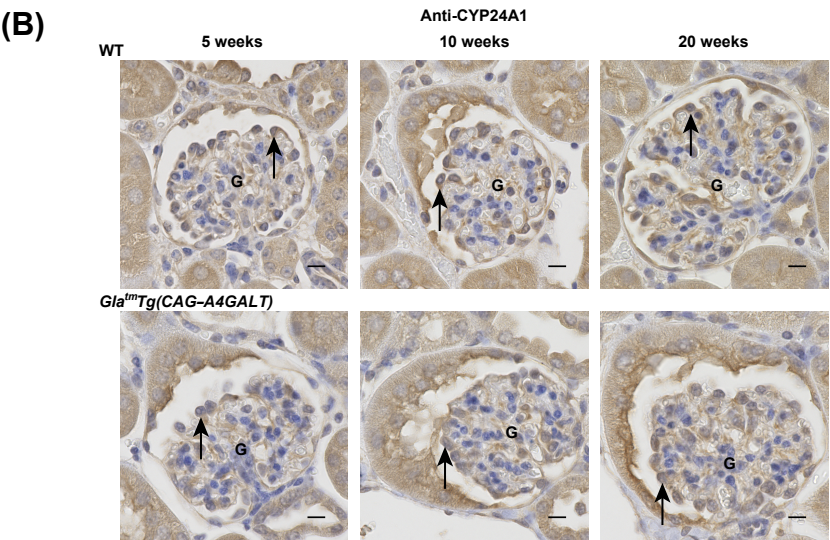

Supplement: Supplementary file 1 — Fig S1‐S3 [file FBA2-2-365-s001.pdf]
