# Supplementary material for: Low bone mineral density due to secondary hyperparathyroidism in the GlatmTg(CAG‐A4GALT) mouse model of Fabry disease
Source: FASEB Bioadv. 2020 Jun 10;2(6):365–81. doi: 10.1096/fba.2019-00080 (PMC7325589; doi:10.1096/fba.2019-00080)
Supplement: Supplementary file 2 — Table S1 [file FBA2-2-365-s002.docx]

**Low bone mineral density due to secondary hyperparathyroidism in the *Gla^tm^Tg(CAG-A4GALT)* mouse model of Fabry disease**

**1. SUPPLEMENTAL TABLE**

**Supplemental Table 1.**

Validation of primary antibodies used for immunohistochemistry and western blotting.

**2. SUPPLEMENTAL REFERENCES**

**3. SUPPLEMENTAL FIGURE LEGENDS**

**Supplemental Figure 1.**

Negative controls for immunohistochemistry.

**Supplemental Figure 2.**

*Gla^tm^Tg(CAG-A4GALT)* mice express 1α-hydroxylase (CYP27B1).

**Supplemental Figure 3.**

*Gla^tm^Tg(CAG-A4GALT)* mice express 24-hydroxylase (CYP24A1).

**1. SUPPLEMENTAL TABLE**

**Supplemental Table 1**. Validation of primary antibodies used for immunohistochemistry and western blotting.

| **Primary antibody** | **Host** | **Source** | **Application (reference*****)** |
| --- | --- | --- | --- |
| **Anti-SLC34A1** | Rabbit polyclonal | Novus  Biologicals  (NBP2-13328) | IHC (S1), WB (S2) |
| **Anti-megalin** | Rabbit polyclonal | Provided by co-author Dr. Saito | IHC (S3), WB (S4) |
| **Anti-CYP27B1** | Rabbit polyclonal | EMD Millipore  (ABN182) | IHC (S5), WB (ND) |
| **Anti-CYP24A1** | Rabbit polyclonal | Aviva Systems Biology  (OABB02030) | IHC (ND), WB (ND) |
| **Anti-F4/80** | Rat monoclonal | AbD Serotec  (MCA497GA) | IHC (S6) |
| **Anti-PTH1R** | Rabbit polyclonal | Merck KGaA  (HPA007978) | IHC (S7) |
| **Anti-GAPDH** | Rabbit polyclonal | Millipore Sigma  (G9545) | WB (ND**^†^**) |

IHC, immunohistochemistry; WB, western blotting; ND, not determined.

*References can be found in the supplemental references.

^†^We searched for articles in the PubMed database that reported validation of this antibody but were unable to find any.

**2. SUPPLEMENTAL REFERENCES**

S1. Robijn, S., Vervaet, B. A., D'Haese, P. C., and Verhulst, A. (2015) Evaluation of intestinal phosphate binding to improve the safety profile of oral sodium phosphate bowel cleansing. *PLoS One* **10**, e0116590

S2. Mace, M. L., Gravesen, E., Nordholm, A., Hofman-Bang, J., Secher, T., Olgaard, K., and Lewin, E. (2017) Kidney fibroblast growth factor 23 does not contribute to elevation of its circulating levels in uremia. *Kidney Int*. **92**, 165–178

S3. Kuwahara, S., Hosojima, M., Kaneko, R., Aoki, H., Nakano, D., Sasagawa, T., Kabasawa, H., Kaseda, R., Yasukawa, R., Ishikawa, T., Suzuki, A., Sato, H., Kageyama, S., Tanaka, T., Kitamura, N., Narita, I., Komatsu, M., Nishiyama, A., and Saito, A. (2016) Megalin-mediated tubuloglomerular alterations in high-fat diet-induced kidney disease. *J. Am. Soc. Nephrol*. **27**, 1996–2008

S4. Hosaka, K., Takeda, T., Iino, N., Hosojima, M., Sato, H., Kaseda, R., Yamamoto, K., Kobayashi, A., Gejyo, F., and Saito, A. (2009) Megalin and nonmuscle myosin heavy chain IIA interact with the adaptor protein Disabled-2 in proximal tubule cells. *Kidney Int*. **75**, 1308–1315

S5. Liu, E., Zalutskaya, A., Chae, B., Zhu, E., Gori, F., and Demay, M. (2014) Phosphate interacts with PTHrP to regulate endochondral bone formation. *Endocrinology* **155**, 3750–3756

S6. Nagase, M., Kurihara, H., Aiba, A., Young, M. J., and Sakai, T. (2016) Deletion of Rac1GTPase in the myeloid lineage protects against inflammation-mediated kidney injury in mice. *PLoS One* **11**, e0150886

S7. Kimura, S., and Yoshioka, K. (2014) Parathyroid hormone and parathyroid hormone type-1 receptor accelerate myocyte differentiation. *Sci. Rep*. **11**, 5066

**3. SUPPLEMENTAL FIGURE LEGENDS**

**Supplemental Figure 1.** Negative controls for immunohistochemistry. Micrographs of *Gla^tm^Tg(CAG-A4GALT)* and WT samples without primary antibody staining for solute carrier family 34 member 1 (SLC34A1), megalin, 1α-hydroxylase (CYP27B1), 24-hydroxylase (CYP24A1), F4/80, and parathyroid hormone 1 receptor (PTH1R). N-Histofine simple stain mouse MAX-PO (R) was used as a secondary antibody for SLC34A1, CYP27B1, CYP24A1, and PTH1R. Biotinylated anti-rabbit IgG was used as a secondary antibody for megalin. N-Histofine simple stain mouse MAX-PO (rat) was used as a secondary antibody for F4/80. Counterstaining with hematoxylin was performed on all slides. Scale bars = 10 μm. mTAL, medullary thick ascending limb; NC, negative control; PCT, proximal convoluted tubule.

**Supplemental Figure 2.** *Gla^tm^Tg(CAG-A4GALT)* mice express 1α-hydroxylase (CYP27B1). *A*, *B*) Micrographs of CYP27B1 expression in the tubules (*A*) and glomeruli (*B*) of *Gla^tm^Tg(CAG-A4GALT)* and WT mice (*n* = 3/group). Scale bars = 10 μm. Arrows indicate CYP27B1-positive podocytes. CD, collecting duct; DCT, distal convoluted tubule; G, glomerulus; PCT, proximal convoluted tubule; PST, proximal straight tubule.

**Supplemental Figure 3.** *Gla^tm^Tg(CAG-A4GALT)* mice express 24-hydroxylase (CYP24A1). *A*, *B*) Micrographs of CYP24A1 expression in the tubules (*A*) and glomeruli (*B*) of *Gla^tm^Tg(CAG-A4GALT)* and WT mice (*n* = 3/group). Scale bars = 10 μm. Arrows indicate CYP24A1-positive podocytes. CD, collecting duct; DCT, distal convoluted tubule; G, glomerulus; PCT, proximal convoluted tubule; PST, proximal straight tubule.
